# Deciphering the role of physical activity in stress management during a global pandemic in older adult populations: a systematic review protocol

**DOI:** 10.1186/s13643-021-01678-6

**Published:** 2021-05-07

**Authors:** Ryan Churchill, Indira Riadi, Lucy Kervin, Kelly Teo, Theodore Cosco

**Affiliations:** Department of Gerontology, Simon Fraser University, Vancouver, V6B 5K3 Canada

**Keywords:** Stress management, Physical activity, Pandemic, Intervention, Older adults, Systematic review

## Abstract

**Background:**

The world has changed dramatically since the beginning of 2020 due to COVID-19. As a result of the pandemic, many older adults are now experiencing an increased and unprecedented amount of psychological stress. Physical activity has been found to be an evidence-based means of combating stress among older adults to promote their quality of life. Studies have demonstrated that those who are physically active experience fewer issues in regard to their mental health, specifically depression and anxiety disorders. Engagement in physical activity may exert a protective influence over stress inducing events and future mental health outcomes. Due to exercise being inexpensive, non-invasive, and effective even via incremental increases in activity level, physical activity interventions should be investigated as a therapy for reducing stress for older adults during the current pandemic.

**Methods:**

Four electronic databases (PubMed, PsycInfo, Web of Science, and SportDiscus) will be searched to identify randomized controlled trials that evaluate the effectiveness of physical activity or exercise programs as a psychological stress management tool in adults 50 years of age or older. Only peer-reviewed and published journal articles will be reviewed. Post-intervention psychological stress measures in comparison to baseline stress will be the primary outcome of interest. All studies will be assessed for bias using Cochrane’s risk of bias tool. A random effects meta-analysis will be investigated if sufficient evidence of homogenous research exists and the heterogeneity of effect sizes will be tabulated.

**Discussion:**

This review will determine the effectiveness of various physical activity interventions for the treatment of psychological stress among the older adult population. This knowledge will help inform care aides, clinicians, family members, and older adults themselves of the most effective physical activity interventions in dealing with stress which is relevant to the ongoing pandemic.

**Systematic review registration:**

PROSPERO CRD42020192546

**Supplementary Information:**

The online version contains supplementary material available at 10.1186/s13643-021-01678-6.

## Background

The world as we know it has changed dramatically since the beginning of 2020, largely impacted by the pandemic caused by the SARS-COV-2 virus, also known as COVID-19. It has had a devastating effect on global economies and health care outcomes (i.e., sickness and loss of life), particularly for the older adult population worldwide [1]. According to the World Health Organization, older adults have an increased risk of developing serious symptoms from the illness [2]. At the onset of the pandemic, multiple stay at home orders were instituted. This prevented meaningful social connections and reduced possibilities of community-based physical activity. With regard to older adult leisure-time activities, increases in television viewing time have been shown to have both negative psychological and physiological manifestations [3]. Before the pandemic, adults aged 65 and older were the highest consumers of cable news [4], and this has been further aggravated by the pandemic. Due to the uncertainty around the pandemic and the increase in time spent indoors, the potential for TV watching and stress is heightened. Due to these  factors and the increased COVID-mortality as it relates to age [1], stress management in the senior population is of utmost importance.

When this pandemic began, older adults were subjected to a novel stress-invoking situation. Guided by Lazarus and Folkman’s transactional theory of stress and coping [5], stress is defined as an imbalance between demands and an individual’s available resources. Stress can occur when the pressure of an event or situation, such as the pandemic, surpasses a person’s capabilities to cope. Researchers have found that stress has a significant negative impact on cognitive abilities in later life [6]. A more dire result of unaddressed stress is suicide. During the SARS epidemic of 2003, there was a substantial spike (32% increase year over year) in suicides for the subgroup of adults aged 65+ in Hong Kong [7]. In an important study looking at the suicide motives of older adults during this time, social disengagement, mental stress, anxiety, and the feeling of being a burden to their families were cited as reasons for the suicides [8]. More importantly for the current pandemic, they recommended that mental and psychological wellbeing be taken into careful consideration when developing epidemic control measures, especially that of older adults. To offset the negative impacts of stress, protective factors need to be explored.One possible cost-effective, non-invasive intervention available to older adults to cope with these circumstances is physical activity. Studies have pointed to physically active people having reduced mental health problems, specifically depressive and anxiety disorders [9]. This evidence points to the wide ranging benefits of physical activity which will benefit many aspects of older adults’ lives. Exercise has been shown to have a protective influence on mental health, specifically in alleviating stress, through the development of resilience. Studies have shown the varying degrees of physical activity intensity in determining negative mood change [10, 11]. This is a key factor for consideration when comparing possible intervention strategies for older adults in that the largest increase in positive effects were seen in the comparison of sedentary individuals and light physical activity (2–4 h per week). Intense activity is not required; gradually increasing baseline activity can be beneficial [10, 11]. This paper will review literature on exercise interventions to explore the optimal type, intensity, and duration of the programs. The level of feasibility of the interventions will also be examined. This review will help inform policy recommendations to support the mental health of older adults as they cope through a pandemic.

## Methods/design

This systematic review protocol has been registered with the International Prospective Register of Systematic Reviews (PROSPERO; Protocol ID: CRD42020192546). Any protocol amendments will be tracked. The Preferred Reporting Items for Systematic Review and Meta-Analysis Protocols 2015 checklist (PRISMA-P) was used to develop this protocol [12] (see Additional file 1).

### Types of studies

Experimental (randomized controlled trial (RCT)) study designs that compare psychological stress levels before and after a physical activity intervention will be examined. Only original peer-reviewed published research will be included. Older studies will not be excluded. Studies written in all languages will be included.

### Types of participants

Studies including older adults of any gender without severe pre-existing medical conditions that could affect their ability to perform the intervention will be included. Studies must include participants aged ≥ 50 years. If studies include multiple age groups, only data from the 50+ age group will be used for the review.

### Types of interventions

This review will include physical activity interventions that include aerobic and/or anaerobic physical activity and can include individual or group-based interventions. Due to the variability of physical activity interventions, it is important that the study in question addresses the intensity—using the Copeland Threshold [13] or other similar intensity thresholds to distinguish between leisure, low, moderate, or vigorous physical activity—and duration of the intervention used. Studies that do not include measurable physical activity outcomes will be excluded.

### Types of comparators

Comparator conditions will include participation in (a) a non-exercise activity or (b) no intervention.

### Types of outcomes

Physical activity must only be assessed using objective methods (e.g., pedometer, accelerometer, HR monitor, V02 Max) and must not be self-reported. As it relates to stress, it is the subjective perception that will be investigated through various psychometric tests or scales (for example, the perceived stress scale (PSS) [14].

### Search methods for the identification of studies

#### Data collection and analyses

The following databases will be searched: PubMed, Web of Science, PsycINFO, and SPORTDiscus.

Keywords will be related to stress management, older adults, and physical activity interventions. Appropriate keywords to identify studies using an experimental and randomized control study design will be employed. One example would be: “Physical activity or exercise” AND “psychological stress or distress” AND “reduc* or control or manag* or prevent*” AND “older adults or seniors or elderly or geriatric” AND “intervention”. For a study to be included, it must include an appropriate control group. References of the included studies will be searched to identify additional potentially relevant studies.

#### Selection of studies

Articles will be imported into Endnote software and all duplicates will be removed. Titles and abstracts will be screened for potential relevance. Full text of the relevant studies identified during previous screening will be reviewed to ensure screening inclusion criteria was met. This screening will be done by two reviewers. Any disagreements will be resolved through discussion and consultation with a third reviewer.

#### Data extraction

This systematic review will conform to the guidelines outlined by the Preferred Reporting Items for Systematic Reviews and Meta-Analyses (PRISMA) checklist [12].

Extracted data will include information on publication (title, authors, year of publication, country of publication), population (participant sociodemographic characteristics and final sample size), intervention type and dosage, control type and dosage, mode of intervention delivery (community centre, laboratory, other setting), type of analysis, outcomes (perceived stress via survey or questionnaire, heart rate, step count, oxygen levels, time intervals, BMI), dropouts, and any adverse effects. Specific characteristics will be collated into a table for the complete review.

#### Assessment of risk of bias in included studies

Risk of bias (ROB) assessment will be appropriately selected depending on the design of the studies included in the final synthesis. ROB will be evaluated both within and across included studies using Cochrane’s risk of bias tool [15]. ROB will be assessed by two review authors, with discrepancies resolved by consensus with a third reviewer.

#### Data synthesis

A formal narrative synthesis is planned and studies that are included will be presented in summary tables with extracted data. Meta-analyses will only be performed when at least three included studies are sufficiently homogeneous in terms of study design, participants, interventions, and outcomes to provide meaningful summary measures. Effect sizes expressed as odds ratios (for categorical data) and weighted mean differences (for continuous data) and 95% confidence intervals will be used for analysis. A random-effects meta-analysis will be performed due to the likelihood of similar effect sizes, but not completely uniform throughout all studies. Heterogeneity will be assessed using the *I*^2^ statistic with values above 75% and *p*< 0.05 used to indicate high heterogeneity [16]. If there is high heterogeneity, a meta-analysis will not be initiated. Where a meta-analysis is not possible, a narrative synthesis will be conducted. The quality of the evidence will be assessed using the GRADE (Grading of Recommendations Assessment, Development and Evaluations) approach [17]. Finally, publication bias will be investigated by charting and deciphering the symmetry of a funnel plot for all studies considered in this review [18].

#### Subgroup analyses

If possible, subgroup analysis will be done to investigate if gender, BMI, or other factors play a role in stress level post-intervention.

## Discussion

To our knowledge, this will be the first review that synthesizes information on the effectiveness of physical activity interventions promoting stress management in older adults aged 50 years and older. Change in stress levels was chosen to address the current pandemic’s strain on older adults and their mental health. We hope to find evidence of the most effective form of physical activity considering variability in the type, duration, and intensity of interventions. This will provide recommendations to guide health practitioners and older adults in choosing the most efficient and effective stress management techniques. The results from the information contained in this review will be distributed via peer-reviewed, open access publication. The review authors will present findings to health care providers and community stakeholders.

## Supplementary Information

**Additional file 1.** PRISMA-P 2015 Checklist.

## Data Availability

Not applicable

## Abbreviations

CDC: Center for Disease Control and Prevention
HR: Heart rate
PRISMA-P: Preferred Reporting Items for Systematic Reviews and Meta-Analyses Protocols
PROSPERO: International Prospective Register of Systematic Reviews
ROB: Risk of bias
VO2 Max: Maximum oxygen volume
WHO: World Health Organization
